# Cognitive reserve and individual differences in brain tumour patients

**DOI:** 10.1093/braincomms/fcad198

**Published:** 2023-07-08

**Authors:** Barbara Tomasino, Gianni De Fraja, Ilaria Guarracino, Tamara Ius, Serena D’Agostini, Miran Skrap, Raffaella Ida Rumiati

**Affiliations:** Scientific Institute, IRCCS E. Medea, Unità Operativa Pasian di Prato, Udine 33037, Italy; Nottingham School of Economics, University of Nottingham, University Park, Nottingham NG7 2RD, UK; CEPR, London EC1V 7DB, UK; Scientific Institute, IRCCS E. Medea, Unità Operativa Pasian di Prato, Udine 33037, Italy; Unità Operativa di Neurochirurgia, Azienda Sanitaria Universitaria Friuli Centrale, Udine 33100, Italy; Unità Operativa di Neuroradiologia, Azienda Sanitaria Universitaria Friuli Centrale, Udine, Italy; Unità Operativa di Neurochirurgia, Azienda Sanitaria Universitaria Friuli Centrale, Udine 33100, Italy; Neuroscience Area, Scuola Internazionale Superiore di Studi Avanzati, Trieste 34136, Italy; Dipartimento di Medicina dei Sistemi, University of Rome ‘Tor Vergata’, Roma 00133, Italy

**Keywords:** cognitive reserve, education, socio-economic status, brain tumour, MRI

## Abstract

The aim of the paper is to determine the effects of the cognitive reserve on brain tumour patients’ cognitive functions and, specifically, if cognitive reserve helps patients cope with the negative effects of brain tumours on their cognitive functions.

We retrospectively studied a large sample of around 700 patients, diagnosed with a brain tumour. Each received an MRI brain examination and performed a battery of tests measuring their cognitive abilities before they underwent neurosurgery. To account for the complexity of cognitive reserve, we construct our cognitive reserve proxy by combining three predictors of patients’ cognitive performance, namely, patients’ education, occupation, and the environment where they live. Our statistical analysis controls for the type, side, site, and size of the lesion, for fluid intelligence quotient, and for age and gender, in order to tease out the effect of cognitive reserve on each of these tests. Clinical neurological variables have the expected effects on cognitive functions. We find a robust positive effect of cognitive reserve on patients’ cognitive performance. Moreover, we find that cognitive reserve modulates the effects of the volume of the lesion: the additional negative impact of an increase in the tumour size on patients’ performance is less severe for patients with higher cognitive reserve. We also find substantial differences in these effects depending on the cerebral hemisphere where the lesion occurred and on the cognitive function considered. For several of these functions, the positive effect of cognitive reserve is stronger for patients with lesions in the left hemisphere than for patients whose lesions are in the right hemisphere. The development of prevention strategies and personalized rehabilitation interventions will benefit from our contribution to understanding the role of cognitive reserve, in addition to that of neurological variables, as one of the factors determining the patients’ individual differences in cognitive performance caused by brain tumours.

## Teaser

Using a large sample, Tomasino *et al.* show that cognitive reserve—proxied by education, professional background, and the nature of their urban environment—helps patients cope with the negative effects of brain tumours on their cognitive functions.

## Graphical Abstract

**Graphical abstract:**
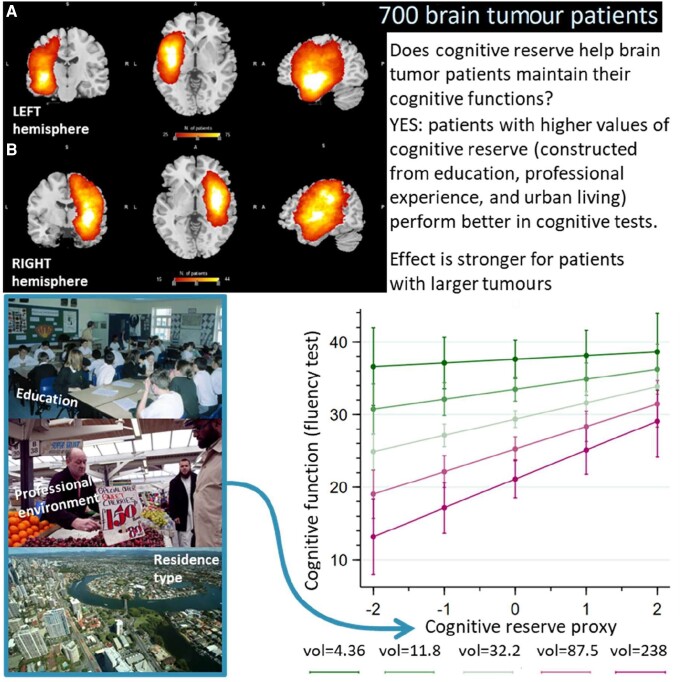

## Introduction

Patients with the same neurological disease and comparable brain damage often display different functional outcomes. For example, more than 25% of elderly individuals with no sign of cognitive impairment met post-mortem pathological criteria of Alzheimer’s disease (AD).^1^ By the same token, 10–40% of individuals with mild to moderate brain pathology showed no clinical symptoms of dementia.^2,3^ To account for this divergence between clinical symptoms and brain damage, the literature has proposed the construct of cognitive reserve (CR).^4–8^ This is defined^6^ as ‘the ability to […] maximize performance through differential recruitment of brain networks [or] alternate cognitive strategies’. The early literature distinguished between the ‘passive models of reserve’ or brain reserve, whereby, for example, ‘individuals with larger brain size or head circumference have less severe AD or are less likely to develop AD’^9,10^, and the active models, like ‘cognitive reserve [which] parallels the concept of brain reserve [as] a potential mechanism for coping with brain damage’ (^6^ p. 451). It is held^6–8^ that ‘An individual who uses a brain network more efficiently, or is more capable of calling up alternate brain networks or cognitive strategies in response to increased demand may have more cognitive reserve’. CR can be proxied by indirect measures,^6^ ‘such as education, IQ, occupation, and lifestyle [which many studies have shown to be] good predictors of which individuals can sustain greater brain damage before demonstrating functional deficit’. CR is effectively a built-in redundancy which affords ‘greater resilience in the face of brain damage’. Stern proposes the illuminating analogy of a ‘trained mathematician [who] might be able to solve a mathematics problem in many different ways, while a less experienced individual might have only one possible solution strategy available. The mathematician would have more flexibility in solving the problem if any particular solution strategy was precluded’ (^6^ p. 452).

The early studies^6–8^ of CR referred mainly to AD but also suggested that other causes of brain damage may also be mitigated by CR.^9,10^ This is indeed confirmed by the a large body of subsequent literature: better education correlates both with a higher level of cognitive functioning and with the delayed onset of cognitive decline,^11^ while low education is a risk factor for dementia and exacerbates its development.^7,12,13^ In addition, a patient’s high level of education has been shown to be associated with less severe post-stroke cognitive deficits^14–16^ and a low level of education, analogously to a low premorbid fluid IQ, increases vulnerability to cognitive impairment after traumatic brain injury,^17–20^ and^21^ for a review.

However, the role of CR in mitigating the neurocognitive dysfunction associated with tumours has been neglected. In this paper, we address this gap in the literature. In addition to education, we included as CR proxies also other factors of interest. The environmental stimuli given by cognitive challenges in the course of professional activities,^22–29^ and cultural stimulation available in large urban centres more than in rural locations,^30–33^ are known to mitigate cognitive decline in aging,^25–27^ multiple sclerosis, or AD.^22–24,28,29^ Accordingly, we construct a measure of CR based on each patient’s education, occupational attainment, and the urban environment of their residence. Previous analyses of neurocognitive dysfunction associated with tumours focused on the effects on cognitive performance of the severity of the tumour, its location, the type of treatment, the age, and the mood of patients. These analyses are typically small observational prospective cohort studies: the interquartile range of the sample size of 23 papers included in a recent meta-analysis^34^ is 19–33. The cognitive functions of patients with high-grade gliomas were found to be more impaired than those with low-grade gliomas, especially for patients undergoing first surgery.^34^ Personal characteristics such as age and mood were found to predict test performance in patients with both low and high gliomas.^35,36^ Brain reserve feature in this literature only in studies the role of IQ, which was found to predict linguistic^37^ and executive^38^ functions in two separate groups of 100 and 166 brain tumor patients respectively. By contrast, we included a large sample of around 700 patients diagnosed with a brain tumour between 2008 and 2019. Our very large sample allows us to study in details novel aspects of the manifold effects of brain tumours on a variety of cognitive functions by considering the role of CR proxies which have been found to mitigate other sources of brain damage, in relation to the neurological variables, such as the site and volume of lesion, hemisphere affected, and histology. Our statistical analysis allows us to study the difference in the effect for right and left hemisphere patients, and possible modulation of CR on the impact of the volume of the lesion. We achieve the latter by including the interaction terms of CR both with the hemisphere and with the volume of the lesion. We also account separately for the various components of CR. Each patient underwent a magnetic resonance imaging (MRI) examination, and their cognitive performance was measured on the same day. The impact of tumours of the central nervous system which develop in the brain on different cognitive functions is known to depend on the location of the lesion and determines a complex link between the extent and nature of brain damage and the severity of the pre-surgery deficit.^34–40^ To account for this complexity, we administered several tests to each patients, selecting those designed to assess the cognitive functions affected by the lesions.

The aim of the paper is to determine the effects of CR on brain tumour patients’ cognitive functions and, specifically, if CR helps patients cope with the negative effects of brain tumours on their cognitive functions.

## Materials and methods

### Subjects: clinical analysis

**Figure 1:**
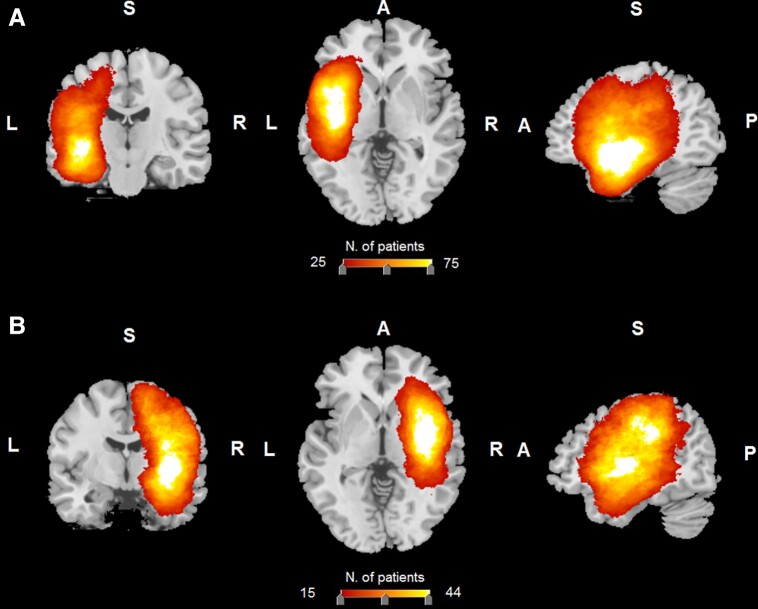
**Brain areas maximally invaded by the tumour in the whole patients’ sample (*N* = 743 lesion volumes).** Brain areas maximally invaded by the tumour in the whole patients’ sample (*N* = 743 lesion volumes) are shown in the overlays of lesion volumes with lesions to the (**A**) left hemisphere, and (**B**) right hemisphere on coronal, sagittal, and axial sections of the T1-weighted MRI template a standard reference for brain MRI. The legend shows the colour corresponding to the different frequencies of the participants’ lesion overlays, from red, least frequent, to white, most frequent.

We retrospectively reviewed a large sample of adult Caucasian patients who were tested before surgery for brain tumour at the Neurosurgery Department of the University Hospital in Udine, in north-east Italy, in the period from 2008 to 2019. We limited our sample for this study to patients whose diagnostic brain MRI showed low- or high-grade glioma, meningioma, metastasis, or cavernoma/arterial–venous malformation, and who had received a neuropsychological evaluation. We included only native Italian speakers, aged between 14 and 80, with normal or corrected-to-normal vision, and no history of previous psychiatric disease or drug abuse. We excluded individuals who reported learning disabilities or a history of developmental language problems. This left a sample of 743, of whom 673 could be included in the analysis as they had all the necessary background information. Table 1 reports the frequency of the diagnoses affecting the patients in the sample used for the research, and Supplementary Table 1 confirms that the 70 individuals who were excluded are not systematically different from those in the sample. The overlay plot of the lesions suffered by all the participants maps the frequency of the damaged voxels in the sample. The legend in Fig. 1 indicates the colour coding of the frequency: the areas in white are those with the worst damage, the left insula, the superior and middle temporal gyri (and temporal pole), the hippocampus and to the right insula, the Rolandic operculum, and the white matter beneath, and in red those least affected. The complete set of MNI coordinates of lesion overlay is shown in Supplementary Table 2.

**Table 1:** Clinical summary statistics

	Left Hem	Right Hem	Total
Low-grade glioma	143	92	235
High-grade glioma	181	110	291
Metastasis	12	5	17
Meningioma	40	27	67
Cavernoma/arteriovenous malformation	29	18	47
Other	12	4	16
Frontal	163	107	270
Fronto-parietal	6	15	21
Fronto-temporal	60	41	101
Occipital/parieto-occipital/temporo-occipital	14	14	28
Parietal	50	41	91
Temporal	101	23	124
Temporo-parietal	23	15	38
Recurrent tumour/relapse	98	71	169
First surgery	319	185	504
Total	417	256	673

Frequency of diagnoses in the patients included in the regression sample, in the left and right hemisphere. Supplementary Table 1 reports summary statistics for the patients excluded and confirms that they are not systematically different from those included.

### Magnetic resonance imaging data acquisition

We used structural imaging data routinely acquired during pre-surgery investigations. A 3-T Philips Achieva whole-body scanner was used to acquire structural data using a SENSE-Head-8 channel head coil and a custom-built head restrainer to minimize head movements. High-resolution T2-weighted and post-gadolinium contrast T1-weighted anatomical MR images were acquired. Structural MRI pre-processing was performed on a UNIX workstation (Ubuntu 8.04 LTS. i386. www.ubuntu.com) using MATLABr2007b (The MathWorks Inc., Natick, MA, USA). Volumes of interest of the patients’ lesions were drawn on their T1 MRI scans using MRIcron software (www.nitrc.org/projects/mricron). We then normalized the ROIs to the Montreal Neurological Institute (MNI) space using the ‘Clinical Toolbox’ (www.nitrc.org/projects/clinicaltbx) for SPM12 (www.fil.ion.ucl.ac.uk/spm), by applying the normalization procedure of the toolbox. Normalized output was visually inspected to rule out inaccuracies in the normalization procedure due to possible mass effects and tissue displacements that lesions can create. The outputs are percentage overlay maps portraying the percentage of overlapping, for each voxel on a colour scale. The study was approved by the local Ethics Committee and carried out in accordance with the 2013 Fortaleza version of the Helsinki Declaration and subsequent amendments. Written informed consent for surgery was obtained. As the study was retrospective, written consent to participate in the study was not applicable.

### Cognitive reserve proxy

To construct our CR proxy, we combine three predictors of patients’ cognitive performance routinely used by the literature, as described above. These are the patients’ education, their occupation, and the geographical setting where they live. Education is measured as the patient’s self-reported number of years spent in formal education. This is the proxy of CR most widely used in studies in normal and pathological ageing^41^ as well as in neurological populations. Occupation is also a patient’s self-reported information, and we followed loosely the UK Office for National Statistics’ occupational classification^42^ to allocate patients in the sample to one of four socio-economic statuses and occupational categories: higher and intermediate white-collar occupations, supervisory and junior white-collar occupations, skilled manual, and semi-skilled and unskilled manual occupations. To these, we have added the further category of pensioners. Given our focus on the stimulus provided by professional activity, pensioners may have more in common with each other than with members of their pre-retirement group, a piece of information which is anyway unavailable in many cases. The third component is included to capture the different cultural environments in different geographical settings: we distinguish whether patients live in a large- or in medium-sized city (over 800 000 inhabitants or between 55 000 and 800,000, respectively), or in a rural environment, that is urban centres with fewer than 55 000 inhabitants. This demographic information for our sample is reported in Table 2.

**Table 2:** Summary statistics for the CR proxies and the demographic variables

	Mean/frequency	SD/percentage	Min	Max
Education (in years)	12.43	3.77	3	19
Urban: large city	55	7.4%		
Medium city	302	40.65%		
Small town/rural	386	51.95%		
SES professional	105	14.27%		
Administrative	250	33.65%		
Manual skilled	174	23.42%		
Manual unskilled	143	19.25%		
Pensioners	70	9.42%		
Composite CR measure	*−0.004*	*1.2*	*−2.71*	*2.87*
IQ: RCPM	28.95	6.63	6	36
Age	46.62	14.46	14	78
Female	338	45.49%		
Left-handed	39	5.25%		
Residence: north	535	72.01%		
Centre	79	10.63%		
South and islands	129	17.36%		
Observations	743			

Summary statistics for the demographic variables used in the regressions. For variables which take many values, we report the mean, the standard deviation, and the lowest and highest values. For binary and categorical variables, we report the frequency and the percentage of the total sample. There are five self-reportedly ambidextrous patients; we have included them in the left-handed group. CR is cognitive reserve; SES is socio-economic status; RCPM is Raven Coloured Progressive Matrices.

**Figure 2:**
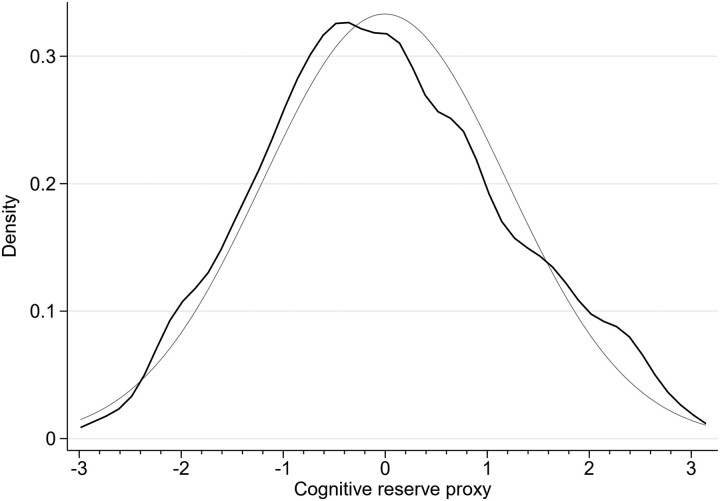
**CR distribution in the sample.** Density of the computed values of CR in the patient’s sample (*N* = 743), drawn as the Gaussian kernel, with bandwidth 0.27. The components of the CR were combined using a principal component analysis of years of education, the five categories of occupational attainment, and the three categories of size of the population centre; we computed the components using polychoric correlations, to account for the categorical nature of the three variables included. The normal distribution with the same mean (0) and standard deviation (1.2) is superimposed as the thin grey line. The lowest and highest values for the CR proxy reported in the data are −2.72 and 2.87.

We combined these components of the CR using a principal component analysis of years of education, the five categories of occupational attainment, and the three categories of size of the population centre; we computed the components using polychoric correlations,^43^ to account for the categorical nature of the three variables we included. The polychoric principal component analysis is calculated using the user-provided command ‘polychoricpca’ in Stata. When used on our data, it computes the relatively moderate correlation measures of 0.24 and 0.56 between the years of education and the size of the population centre and the five occupational categories, respectively, and of 0.27 between the size of the population centre and the five occupational categories. The largest eigenvalue is 1.73. The distribution of this measure in the sample is reported in Fig. 2. We have superimposed the normal distribution with the same mean and variance as the actual distribution in our sample.

### Other control variables

Our empirical analysis also controls for variables which, in theory, may also affect the patients’ performance in the cognitive performance tests. Clearly, one of them is fluid intelligence, operationalized by IQ, as the foundation component of CR, upon which education and other environmental influences build.^41,44^ We measure it by the patient’s performance on a non-verbal intelligence test, the Raven Coloured Progressive Matrices (RCPM).^45^ We also control for the patient’s age, including a quadratic term to capture possible non-linearities, and their gender. We distinguish between the broad areas of Italy where the patient resides: north, centre, and south and islands. Finally, we include the date when the test was performed, to account for possible trends in the experience of the team administering it.

### Cognitive assessment

Patients underwent the neuropsychological assessment on the same day of the MRI examination. This consisted of ten tests, assessing various functions. A summary of the performance in these tests is presented in Table 3, which also contains summary statistics for the volume of the tumour. The memory tasks are the working memory (WM) tasks,^46,47^ which tests their ability to retain and recall items during reasoning, comprehension, and learning, and the short-term memory (STM), which measures their ability to maintain for a short period of time verbal or spatial information active in memory. Executive functions were measured by fluency tasks.^48,49^ For these three tests, patients with left hemisphere lesions performed the verbal version and those with right hemisphere lesions the visual version. We measure cognitive flexibility by using the time taken to complete Trail Making Tests A and B,^50^ measuring psychomotor speed and selective attention (TMT-A), attention shifting (TMT-B), and cognitive flexibility (TMT-B-A), given by the difference between the score in the TMT-B and the TMT-A. TMTs are measured in time, so, unlike the other tests, a lower value indicates better accuracy.

**Table 3:** Summary statistics volume and cognitive performance

	Full sample		Left hemisphere		Right hemisphere	
	Mean	SD	Mean	SD	Mean	SD
Volume	52.488	47.838	49.118	42.669	57.978**	54.88
STM	5.24	1.17	5.324**	1.268	5.077	0.933
WM	3.918	1.165	3.739	1.193	4.271***	1.021
Fluency	29.37	14.322	30.087**	15.112	25.975	9.072
TMT-A	41.568	27.923	44.969*	30.906	39.618	25.93
TMT-B	119.311	71.704	133.423**	73.801	111.349	69.405
TMT-B-A	79.218	56.952	91.131**	59.561	72.55	54.447

Test for left hemisphere			Test for right hemisphere			
	mean	SD		mean	SD	
Comprehension	32.335	5.719	Construct	13.014	2.098	
Naming	27.063	5.382	Clock	8.944	2.129	
			Cancel	52.331	3.77	

Volume is measured in mm^3^. STM, short term memory; WM, working memory; TMT, trail making test; Comprehension, verbal comprehension; Construct, constructional apraxia; Naming, noun naming; Cancel, cancellation test. Stars after the mean in the left or right hemisphere column indicate a larger value for the corresponding hemisphere, with statistical significance measured by a *t*-test, whose *P*-values are reported by the asterisks: ***P < 0.01, **P < 0.05, *P < 0.1.

Patients with left hemisphere lesions also performed a verbal comprehension task (Compr),^51^ in which they are asked to carry out an action using coloured tokens placed before them, in response to the examiner’s verbal commands, and a task which required them to name the object pictured in a black and white line drawing (Naming).^52^ Patients with right hemisphere lesions completed instead a figure copying task to capture the presence of constructional apraxia (Construction),^53^ the clock drawing test to evaluate visuo-spatial planning abilities (Clock),^54^ and a target cancellation test to measure visuo-spatial attention (Cancel).^55^ Summary statistics for the patients’ performance in these tests are collected in Table 3, which also reports the volume of the lesion. On average, patients affected in the right hemisphere have an 18% larger lesion compared with left hemisphere patients [right hemisphere: mean 57.98 (SD 54.88) versus left hemisphere: mean 49.12 (SD 42.67)]. All cognitive performances differ in a statistically significant way in the two groups of patients (left versus right), though not in the same direction: the WM (*P* < 0.001) and the TMTs (*P* < 0.05) are on average worse for patients with left hemisphere tumours, even though they have smaller lesions, and, conversely, patients with right hemisphere tumours perform worse in STM (*P* < 0.05) and fluency tests (*P* < 0.05).

### Statistical analysis

The data was analysed with 11 multivariable OLS regressions (performed in Stata), 1 for each of the cognitive outcomes as the dependent variable. The independent variables, or covariates, are treated either as continuous—CR, age, the volume of the lesion (in logs), and IQ—, or as categorical variables—left or right hemisphere, histology, tumour site, left-handedness, first occurrence or relapse, residence, and gender. We normalized the continuous variables so that their mean was zero and so the ‘Base level’ row represented the value predicted by our regression for a patient with the average value of the continuous variables, and the values of the categorical variables reported in the note. Statistical significance was assessed with a *t*-test, with thresholds at *P <* 0.01 (***), *P <* 0.05 (**), and *P <* 0.1 (*). The specifications in the regressions are all identical, except for the obvious difference of not including the right hemisphere indicator variable for the test performed on one hemisphere only. All independent variables enter linearly, with the exception of the interaction terms between the CR and right hemisphere, and age, for which we include a quadratic term to account for different effects for young or elderly patients.

### Fluid intelligence proxy

To identify the role of CR as an active brain reserve exerting a role on the maintenance of cognitive capacity separate from that of brain reserve, it is important to confirm that the RCPM test, which is taken on the same day as the MRI examination that confirmed the presence of the tumour, is unaffected by the tumour itself. In other words, we need to verify that the tumour harms the cognitive performance as measured by the cognitive tests but does not affect our measure of fluid intelligence at the foundation of this performance. This is indeed the case for the patients in our sample: Table 4 shows that the test score is not associated with the characteristics of the tumour and by the extent of the lesion (the complete set of coefficients is in Supplementary Table 3). We note two exceptions: relapsed patients achieved a lower score in the RCPM tests, as did those whose tumour is in the parietal region of the brain, though both effects are not strongly statistically significant (*P* < 0.05 and *P* < 0.1, respectively). As one expects, IQ is strongly correlated with education, either because the latter enhances it^37,39^ or because, on average, people with a higher IQ are more likely to stay in education for longer, or both. By the same token, being unskilled is associated with lower RCPM test results.

**Table 4:** Determinants of RCPM test

Variables	Coefficient	SD	*P*-value
Base	0.00591	(0.141)	0.967
Education (years)	0.0614***	(0.0142)	1.73*e*^−05^
Left hemisphere	−0.133	(0.0784)	0.0908
Extra effect for RH	−0.0293	(0.0204)	0.150
Professional	0.158	(0.145)	0.277
Administrative	−0.0768	(0.107)	0.473
Unskilled	−0.258**	(0.116)	0.0269
Pensioners	0.191	(0.164)	0.244
Volume (in log)	−0.0345	(0.0352)	0.328
High-grade glioma	−0.0310	(0.0939)	0.741
Metastasis	−0.223	(0.246)	0.364
Meningioma	−0.0863	(0.150)	0.565
Cavernoma/AVM	−0.117	(0.159)	0.462
Other histology	−0.0422	(0.253)	0.868
Relapsed	0.202**	(0.0907)	0.0262
Fronto-parietal	0.349	(0.217)	0.109
Fronto-temporal	0.119	(0.114)	0.297
Occipital/P/T	−0.169	(0.191)	0.377
Parietal	−0.224*	(0.117)	0.0565
Temporal	−0.00763	(0.109)	0.944
Temporo-parietal	−0.162	(0.167)	0.333
Lefthanded	0.228	(0.173)	0.188
Large city	0.00847	(0.151)	0.955
Smaller city	0.0305	(0.0805)	0.705
Observations	*673*		

Association between the RCPM (Raven Coloured Progressive Matrices) test and characteristics of the tumour. We control for the socio-demographic variables. Included in the regression, age, age squares, gender, and place and nature of the residence, none of which are associated with the test. The base level (top row) is the predicted value of a right-handed male patient of average age and education, with the average RCPM score, at the first surgery for an average log–volume frontal low-grade glioma tumour in the right hemisphere, who is employed as a skilled manual worker or equivalent occupation and lives in a rural location in the north of Italy. AVM is arterial–venous malformation, RH is the right hemisphere, and occipital/P/T is parietal/temporal. The *P*-values are denoted by asterisks: ***P < 0.01, **P < 0.05, and *P < 0.09.

## Results

**Figure 3:**
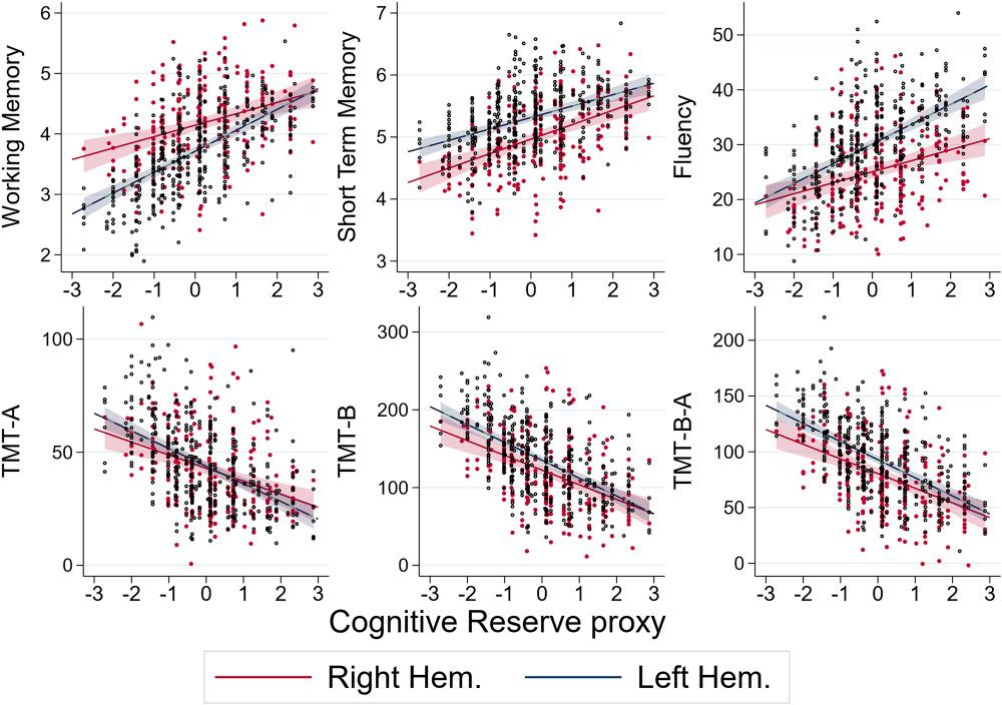
**Predicted cognitive test performance as a function of CR.** In each diagram, a dot represents the value of test for the cognitive function labelled on the vertical axis (WM, STM, fluency, TMT-A, TMT-B, TMT B-A, respectively) predicted by the regression given the values of the patient’s characteristics other than the CR proxy, which is measured on the horizontal axis. The solid lines are the best fit lines with 99% confidence intervals depicted as shaded areas for the six tests performed by both right (coloured in red) and left (navy-blue) hemisphere patients. The sample size for each regression is, from the top left, *N* = 538, *N* = 571, *N* = 459, *N* = 354, *N* = 341, and *N* = 340. The *P*-values for the values of the statistical significance of the coefficients are reported as the number of stars after the coefficient in the third and fourth rows of the first six columns of Table 5: ***P < 0.01, **P < 0.05, and *P < 0.1.

**Figure 4:**
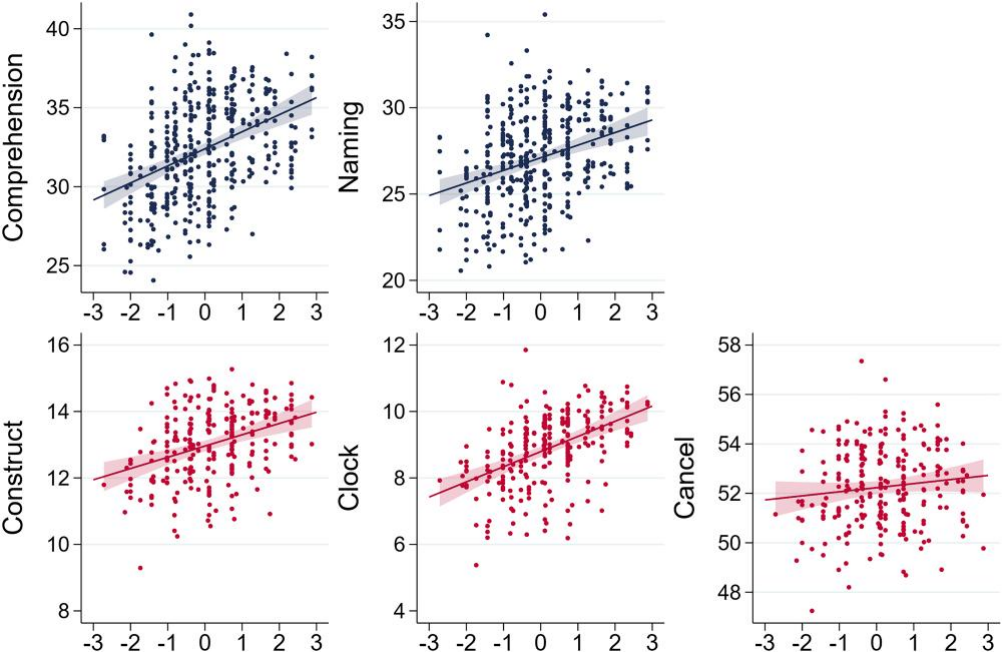
**Predicted cognitive test performance as a function of CR.** As in Fig. 3, dots represent the predicted values of patients’ cognitive function tests (comprehension, naming, constructional apraxia, clock test, and cancellation, respectively) labelled on the vertical axis predicted by the regression given the values of the patient’s characteristics other than the CR proxy, measured on the horizontal axis. The solid lines are the best fit lines with 99% confidence intervals depicted as shaded areas for the two tests performed by left hemisphere patients (top row, coloured in navy-blue) and the three tests performed by right hemisphere patients (bottom row, coloured in red). The sample sizes for the regressions are, clockwise from the top left, *N* = 400, *N* = 416, *N* = 250, *N* = 233, and *N* = 169. The *P*-values for the values of the statistical significance of the coefficients are reported as the number of stars after the coefficient in the third row of the last five columns of Table 5: ***P < 0.01, **P < 0.05, and *P < 0.1.

**Figure 5:**
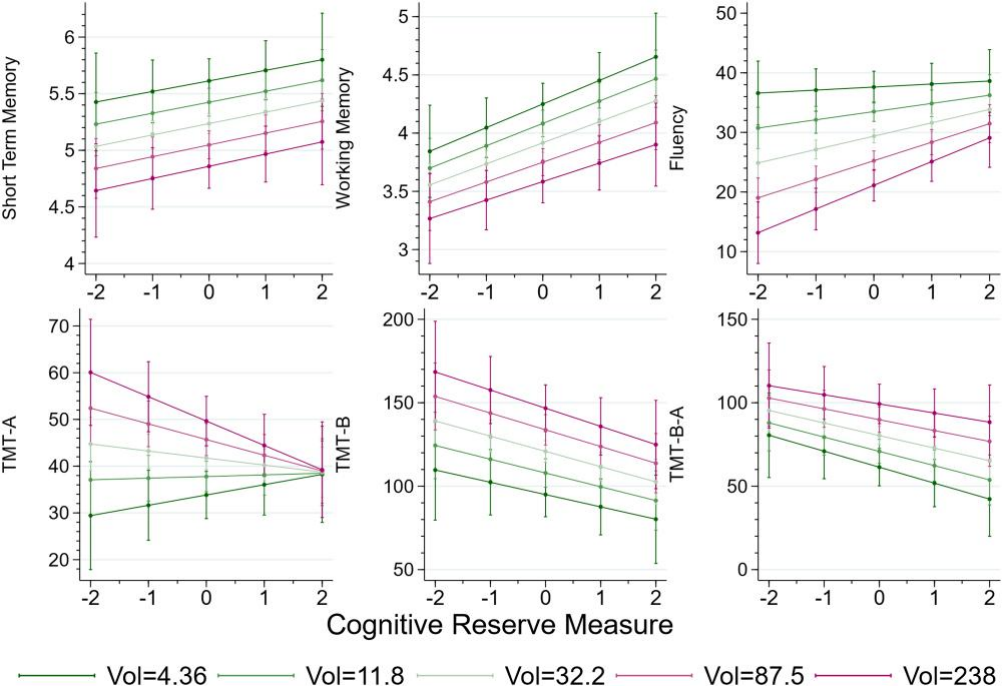
**CR modulation of the effect of the volume of the tumour on cognitive performance.** Each diagram shows the predicted performance for the reference patient whose value of the controls, except CR and volume, as the CR varies, for five values of the tumour size, with green lines indicating a smaller tumour. Each line represents the prediction for one value of the volume of the lesion, as indicated in the legend, from green (smallest) to purple, (darkest). The values correspond to the normalized values of the log volume, from −2 to 2. The sample sizes for each cognitive tests are given in the note to Fig. 3: from the top left, *N* = 538, *N* = 571, *N* = 459, *N* = 354, *N* = 341, and *N* = 340. The statistical tests are given at each level, of the volume: they are represented by the vertical bars which indicate the 95% confidence intervals for the five selected values of the CR. Thus, for example, the fact that in the top right figure, the light-green line (that for patients with volume = 32.2) touches neither the line above nor the line below, indicates for patients whose CR = −1, the model predicts a value of the fluency test such that a *t*-test of the difference of the score predicted for the same test for patients with volume = 11.8 being different from zero would return *P* < 0.05. Similarly for the difference with a patient with volume 87.5.

**Figure 6:**
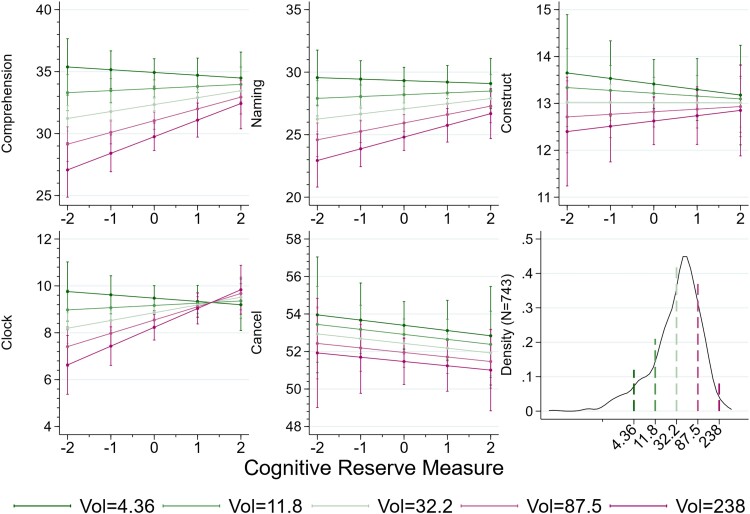
**CR modulation of the effect of the volume of the tumour on cognitive performance.** As in Fig. 5, each diagram shows the predicted performance for the reference patient whose value of the controls, except CR (cognitive reserve) and volume, as the CR varies, for five values of the tumour size, with green lines indicating a smaller tumour. The values correspond to the normalized values of the log volume, from −2 to 2. The sample sizes for each cognitive tests are given in the note to Fig. 4: from the top left, *N* = 400, *N* = 416, *N* = 250, *N* = 233, and *N* = 169. The vertical bars indicate the 95% confidence intervals for the five selected values of the CR, as in Fig. 5: see the note to that figure for an example. The last panel in the bottom row is the density of the volume, in a log scale on the horizontal axis, with the vertical bars indicating the position of the five values used as references in the other panels and in Fig. 5. The sample size for the density is *N* = 743.

The main aim of the paper is to determine the effects of CR on brain tumour patients’ cognitive functions and, in particular, to determine if CR helps patients cope with the negative effects of brain tumours on their cognitive functions. Table 5 and Figs 3–6 below summarize our main empirical findings. To allow for comparisons with previous clinical studies on patients with brain tumour, we begin by reporting the description of the role of neurological variables, the histology, and the site of the lesion. The role of volume and the hemisphere where the tumour is located are discussed below in connection with the analysis of the effects of CR. Regarding the other neurological variables, we find that this is indeed the case: all the detailed results are reported and commented in the online Supplementary material. Supplementary Table 4 shows that when everything else is the same, the cognitive functions of a patient affected by low-grade glioma are less severely affected than a patient affected by other types of tumour, such as high-grade glioma, cavernoma, or arteriovenous malformation.

**Table 5:** CR and cognitive functionality

	(1)	(2)	(3)	(4)	(5)	(6)	(7)	(8)	(9)	(10)	(11)
Variables	WM	STM	Fluency	TMT-A	TMT-B	TMT-BA	Compr	Naming	Construct	Clock	Cancel
Base level	3.860***	5.590***	33.45***	34.38***	102.7***	69.16***	33.91***	28.65***	14.06***	9.488***	53.64***
	(0.134)	(0.144)	(1.776)	(4.393)	(11.35)	(9.552)	(0.757)	(0.737)	(0.379)	(0.422)	(0.929)
Right hemisphere	0.489***	−0.273***	−4.774***	−2.181	−14.59**	−13.77**					
	(0.0913)	(0.0984)	(1.710)	(2.823)	(7.338)	(6.192)					
CR	0.230***	0.0872*	2.584***	−3.009	−11.67**	−9.177**	0.558**	0.410*	−0.00259	0.331**	−0.254
	(0.0464)	(0.0507)	(0.587)	(1.855)	(4.802)	(4.051)	(0.233)	(0.226)	(0.129)	(0.139)	(0.306)
Extra effect for RH	−0.146*	0.0390	−1.959	2.372	3.976	2.580					
	(0.0781)	(0.0844)	(1.343)	(2.228)	(5.847)	(4.923)					
Volume (in log)	−0.167***	−0.189***	−4.122***	3.955***	12.94***	9.479***	−1.296***	−1.129***	−0.197*	−0.311***	−0.482*
	(0.0411)	(0.0441)	(0.607)	(1.157)	(3.058)	(2.576)	(0.254)	(0.244)	(0.115)	(0.119)	(0.278)
Volume × CR	−0.0110	0.00355	0.868*	−1.854*	−0.878	1.023	0.391*	0.264	0.0577	0.236**	0.0124
	(0.0370)	(0.0400)	(0.495)	(1.041)	(2.707)	(2.279)	(0.204)	(0.198)	(0.107)	(0.112)	(0.265)
RCPM score	0.0415***	0.0409***	0.252***	−1.609***	−3.673***	−2.460***	0.165***	0.141***	0.0691***	0.0580**	0.0110
	(0.00851)	(0.00887)	(0.0934)	(0.250)	(0.666)	(0.561)	(0.0446)	(0.0422)	(0.0198)	(0.0233)	(0.0434)
Observations	538	571	459	354	341	340	400	416	250	233	169
*R*-Squared	0.355	0.206	0.295	0.366	0.376	0.300	0.306	0.236	0.249	0.233	0.166

Standard errors in parentheses. The *P*-values report the results of the *t*-test for statistical significance of the difference between the reported coefficient and 0 and are denoted by asterisks: ***P < 0.01, **P < 0.05, and *P < 0.1. The base level (top row) is the predicted value of a right-handed male patient of average age and education, with the average RCPM score, at the first surgery for an average log–volume frontal low-grade glioma tumour in the right hemisphere, who is employed as a skilled manual worker or equivalent occupation and lives in a rural location in the north of Italy. CR is cognitive reserve, RH is right hemisphere, and RCPM is Raven Coloured Progressive Matrices. The table contains only the coefficient of interest; the complete set is available in the online Supplementary tables.

Lesions in the frontoparietal regions have a significant impact on attentional tasks (the TMT-A, either in the right or left hemisphere), while lesions in the same regions of the right hemisphere affected performance on constructional apraxia. Lesions in the right parietal regions produce a selective decline of visual–spatial planning, the clock task, and lesions of the temporal, and temporo-parietal regions reduce patients’ performance on naming, and comprehension in the left hemisphere, and in construction in the left hemisphere for the temporo-parietal regions. Lastly, lesions of the occipito/parietal/temporal regions reduce patients’ performance on attentional tasks either in the right or in the left hemisphere, while lesions in the same regions of the right hemisphere affected performance on constructional apraxia.

We find no additional effect for a relapse on any of the cognitive tests.

Table 4 shows that, naturally, an extra point at RCPM, relative to the RCPM score of the average patient, improves performance in all the tests: it increases WM, STM, fluency, attention, comprehension, naming, construction, and clock.

### Cognitive reserve proxy

Table 5 and Figs 3–6 collect our main statistical results. They highlight the main finding of the paper that CR has an important role in explaining individual differences in the cognitive performance of patients with a brain tumour and specifically that it helps patients maintain neuropsychological abilities when attacked by a brain tumour. The table and the figures evidence substantial differences for left and right hemisphere patients both in the functions influenced by CR and in the magnitude of CR effects. To a less precise extent, the table also shows evidence that CR modulates the effects of the size of the tumour. In Table 5, the *P*-values of the coefficients are indicated by the number of stars, as explained in the note, and the standard errors are reported below the corresponding coefficient, in brackets. Table 5 reports the main coefficient of interest. The complete set of coefficients, that is all the controls that may in principle affect the coefficient of CR but are outside the focus of the paper, is in Supplementary Table 4.

While the main aim of the paper is to determine the effects of CR on brain tumour patients’ cognitive functions, specifically to determine if CR helps patients cope with the negative effects of brain tumours on their cognitive functions, it may also be of peripheral interest to identify the effect of the separate role each component exerts. The large dataset at our disposal allows us to include the three components of the CR separately in our statistical analysis: when we do so, their positive effect on cognitive function is confirmed (see Supplementary Table 5) and further research will be necessary to determine which acts on which cognitive outcome and which exerts positive effects on its own and in combination with other components.

In view of the fact that we have included the interaction term of CR both with the hemisphere and with the volume of the lesion, the coefficients for these variables are not straightforward to interpret on their own and should instead be studied as a set, in conjunction with the graphical analysis presented in the figures.

This different effect of CR on right- and left-hemisphere patients is illustrated in the plots of Fig. 3: in each plot, the dots are the fitted values of each cognitive performance as a function of the patient’s CR for a patient whose other variables are at the average or reference levels, and the straight lines the best fit regression lines; the shaded areas around the line are the 99% confidence intervals. The dot for a specific patient would sit on the line if that patient had all the co-variates other than CR at their sample average or reference value. With the exception of the short memory test, the red lines, for right hemisphere patients, are flatter than the navy-blue lines, the left hemisphere patients, in a statistically significant manner for the WM (coefficient −0.146, *P* ≈ 0.06). This indicates that the beneficial effect of CR is stronger (STM excepted) for patients with a left hemisphere. In the case of WM, the disadvantage that left hemisphere patients have at the lowest CR measure (e.g. those with very low education levels) is indeed completely compensated for those with the highest recorded CR, corresponding to 15 or more years of education. The effect is somehow reversed for fluency: in this case, low CR patients experience approximately the same decrease in performance but CR has a stronger effect for those with a left hemisphere lesion, so that for the higher values of CR, they perform significantly better in the STM test than those affected by a tumour in the right hemisphere.

The TMTs show essentially no difference between the two hemispheres. Finally, patients with a left hemisphere lesion have better cognitive functionality, measured by the STM, as shown by the navy-blue line being above the red one in the middle plot of the top row in Fig. 3, but a given increase in CR has the same effect on the two groups of patients.

Figure 4 shows similar differences in tests that are administrated only to patients with a lesion in one or the other hemisphere.

Figures 5 and 6 are analogous to Figs 3 and 4: the horizontal and vertical axes measure the CR and the performance in each of the cognitive tests we performed, and the separate lines the predicted values of the test for the reference patient, that is one whose characteristics are those described in the notes to Table 5, except for the volume of the lesion, which instead takes the values reported in the legend. These values correspond to the average volume, the thin central line, and volumes corresponding to −2, −1, 1, 2, after normalization of the average log volume to 0. There are however important differences. In the first place, we represent the confidence intervals only with whiskers for the values of the (log) volume which as labelled on the horizontal axis: as the table makes it clear, many of the *P*-values for the volume × CR interaction coefficients point to a lack of statistical significance, and so, the confidence bands would be overlapping and reduce the clarity of the figure. This lack of precision in the estimated coefficients and so the indications of this figures should be taken with a pinch of salt. Nevertheless, the consistency in the direction of the interaction suggests that a larger sample would improve the precision and so confirm the overall gist of our results. This can be summarised by noting that CR modulates the effect of a larger volume, by protecting better patients with a larger tumour for the fluency test, the TMT-A, the Comprehension test, the naming test, the constructional apraxia, and the clock tests, but has no offer of protection against a larger tumour for the remaining tests. It would be naïve to interpret the first figure in the bottom row as suggesting that patients with a low lesion volume are harmed by CR. The positive slope of the green lines in this diagram is an artificial consequence of the constraint that imposes a linear effect of CR on cognitive performance, and a simple multiplicative effect between CR and (log) volume. Were it available, a larger dataset would permit a richer regression analysis, allowing us the better to map the nuance of the role of CR for different patients, instead of the ‘one size fits all’ approach of this paper. Similarly for the Clock test, a naïve interpretation of the figure would lead one to the absurd conclusion that a larger tumour has less negative effect for patients with high values of CR. A less mechanical interpretation is that CR offer protection against the damaging effects of a larger tumour.

With the exception of TMT-A, the test for construction, and the cancellation tests, all other cognitive tests confirm the positive effect of CR, but not its differential effect for right hemisphere patients.

We include interaction terms with CR, in order to identify possible modulation of clinical variables with CR: in particular, as Table 5 shows, patients’ cognitive response to differences in both the hemisphere where the tumour is located and in the volume of the tumour itself varies with their CR. While, as one would expect, a larger lesion is more damaging, it modulates the positive effect of CR, only in some tests, the fluency test, TMT-A, the comprehension test, and clock test, where the negative effects of a larger tumour are somehow tempered by the patient’s CR.

We also performed the analysis by including a triple-interaction term: CR × volume × left hemisphere. This would allow us to identify whether any modulation on the role of volume determined by CR differs in patients with left and right hemisphere lesions. The results are not suggestive of large difference, although this may be due to the fact that, despite its large size, the sample remains unable to detect such non-linear nuances in the nature of the overall effect. We report the results in the online Supplementary Table 6.

## Discussion

CR is a multicomponent construct that has been previously found useful in accounting for how individuals cope with normal and pathological ageing. In this paper, we aim to determine the effects of CR on brain tumour patients’ cognitive functions, specifically to determine if CR helps patients cope with the negative effects of brain tumours on their cognitive functions. In practice, we achieved our aim by investigating whether different values of CR in a large sample of brain tumour patients, with otherwise identical neurological factors and other characteristics, are useful to understand any differences in their cognitive performance.

### The impact of neurological factors on patients’ performance

The neurological factors, volume, hemisphere, region affected, first surgery or relapse, and histology, used as predictors of patients’ cognitive performance, have all their expected effects, as evidenced in the complete tables of the regression coefficients reported in the online Supplementary material. All these results are in line with the existing literature on brain damage. First, the volume of the lesion impacts negatively all tests except for constructional apraxia. The larger the volume of the lesion caused by the brain tumour, the more severe its effect on the patients’ cognitive performance.^56–59^ The complementary evidence is represented by patients with small lesions caused by incidental low-grade gliomas having a small impact on their cognitive abilities.^60,61^

Second, relative to tumours in the left hemisphere, tumours in the right hemisphere weaken patients’ cognitive performance more, particularly on STM and fluency tasks. Moreover, relatively to tumours in other regions, those in the fronto-parietal and temporo-parietal regions have a detrimental effect on more tests. Although the lesion overlay analysis shows that the sites were equally distributed in the two hemispheres (see Table 3), the average volume of tumours in the right hemisphere is 18% larger than that in the left hemisphere and this might explain the more severe deficit of right brain–damaged patients.

Third, we find no additional effect of a relapse on cognitive performance, in line with other work which shows that repeated glioma surgery does not cause major damages to cognitive functions in the 4 months after surgery.^62^ Fourth, in line with previous observations, we find that high-grade gliomas impair performance significantly more than low-grade gliomas.^63–66^ This is likely due to tumour-related molecular determinants, as shown in a study where gene set enrichment analysis was performed to test oncogenetic markers for cognitive impairment; this study found that the molecular characteristics of glioma can be independent determinants of patients’ cognitive functioning.^67^ Among our novel findings, cavernomas or arteriovenous malformations (AVM) have a worse impact on our patients’ cognitive functions than low-grade glioma. This tallies with the suggestion that AVM affect the whole brain networks and not just the area surrounding the lesion, which was established in a resting-state study, whose AVM patients showed altered functional connectivity,^68^ including patients whose lesion was distant from the principal nodes of the default mode network.^68^ In addition, bleeding in the cavernoma has been shown to disrupt the white matter, and thus, it could limit pre-operatory plasticity,^69^ while in the AVM, it is the mechanism of vascular theft which may cause white matter hypoperfusion. The pathophysiology with white matter destruction and also the fact that AVM is an acute event, which does not give time for reorganization and adaptation, explain the observation that those affected by it display a worse performance than those affected by a slow-growing tumour such as a low grade glioma.

### IQ and CR protect patients’ cognitive performance

The novelty of this paper is the study of the role of CR in explaining the effects of brain tumours on patients’ cognitive performance. Our main finding is that, controlling for neurological characteristics, IQ, CR affect strongly a patient’s extent of cognitive impairment following a brain tumour.

The RCPM, a test that returns an IQ as a measure of fluid intelligence, turns out to be a strong predictor of patients’ performance. We find that, while the tumour harms performance on cognitive tests, it does not hamper this measure of fluid intelligence. This is consistent with previous studies which found no significant differences in both pre-surgery RCPM scores between the tumour group and the control groups,^70^ and in the median pre- and post-operative RCPM scores in 43 patients with brain tumours.^71^ In contrast, however, there is evidence that a brain lesion might differentially affect RCPM performance:^72^ the performance on the first set of RCPM, which required reasoning on the identification of sameness, was particularly impaired in right brain–damaged patients, while performance on the second set of items, triggering symmetry-related operations, was altered in aphasic left brain–damaged patients and, lastly that the third set of RCPM, requiring analogical and conceptual thinking, was failed by left brain–damaged patients with or without aphasia. In another work, on tumour patients with parietal lesions, performance was less accurate in RCPM than in making temporal rule inductions, which frontal patients found more challenging.^73^ In line with these works, we do find one small effect of the characteristics of the tumour: patients whose tumour is in the right parietal region of the brain achieve a lower score in the RCPM. This may be due to the fact that some visuo-spatial operations are required to carry out the RCPM task and that such operations are relying on the parietal lobe. Finally, we find the RCPM test is lower in relapsed patients.

CR, which we compute as a weighted average of education, occupational attainment and status, and urban environment, mitigates the effects of the brain tumour on the patients’ cognitive performance. While the protective role of education is well documented in other neurological disorders such as Alzheimer’s disease, vascular dementia and non-specific dementia,^1–4,6,9–13,74,75^ stroke,^14–16^ TBI,^17–21^ and multiple sclerosis,^22^ there is little evidence that it might work also for brain tumour. Higher levels of education seem to positively influence post-surgery cognitive rehabilitation,^76^ to improve patients’ brain network efficiency,^77^ and to influence the relationship between executive function impairment and frontal lesions.^78^ In two studies, with considerably smaller samples than ours, education failed to predict patients’ performance.^37,78^ Our finding is that CR has substantially stronger effects on left hemisphere patients. These differences spur further study of cognitive functions located in the right hemisphere which have hitherto been poorly examined in brain tumour patients.

We include the type of employment and the urban environment in our CR proxy, and, in Supplementary Table 5, we show that they have an additional effect when kept separate from education. The former constitutes an environment-related stimulation, requiring learning and maintaining routines, and this in turn influences CR and promotes *plasticity*.^79^ The nature of a person’s employment has been reported to influence cognitive performance in pathological populations such as those with HIV,^80^ mild cognitive impairment,^81^ relapsing remitting multiple sclerosis,^82^ and AD.^83^ The effects of occupational attainment are observed in healthy ageing.^84–86^ Residence is shown to matter in normal populations: living in an affluent neighbourhood promotes CR in adult ageing.^33,87,88^

It is instructive to quantify the effect of CR on improving performance. To this aim, we can compare a patient with the average value of CR and the average or reference values of all the other variables, with a patient with all the same values of the variables but a measure of CR one standard deviation higher, which would put the latter at around the eighth decile of the CR distribution, rather than the average. We find that the WM is 0.27 (≈1.2 × 0.23) higher in this patient, this is almost one-quarter of the WM standard deviation. This effect is lower in patients with a right hemisphere lesion: while they fare better than their counterpart with a right hemisphere lesion, as shown by the positive coefficient of 0.498 in the first column of Table 5 and the higher intercept range of the red curve in the first diagram of Fig. 3, they benefit less from their CR, as indicated by the statistically significant coefficient of −0.146 and the shallower positive slope of this red curve relative to the blue one; the same increase in CR would increase the average right hemisphere patient’s performance by 0.1 (≈1.2 × (0.23–0.146)), around 8% of the WM standard deviation. To put these figures into perspective, this is equivalent to the increase in WM determined by a whole standard deviation in the RCPM score and suggests that one standard deviation higher value of CR compensates, on average, for a tumour approximately 7.2% larger in volume: measuring the volume in logs means that the coefficient is 100 times the effect of an increase in 1% in volume, and 7.2 ≊ 1.2/0.167.

### CR on brain tumour patients

Summing up, we show that CR proxies can explain individual differences in patients’ cognitive performance not only in pathological ageing, stroke, and TBI but also in brain cancer, further reinforcing the added value of investigating the cognitive functions of patients with brain tumours. Our study is thus in line with the recent attempts of neuropsychologists to study brain tumour patients as a method to uncover neurocognitive mechanisms.^89,90^ In recent years, the neuropsychological deficits often observed in vascular patients have been replicated with some differences from brain tumour patients.^89–99^ Notably, the deficits in tumour patients tend to be milder or to give rise to less dramatic dissociations. As an example, within the language domain, one of us reported the case of an aphasic patient who developed (reproduction) conduction aphasia following a lesion in the Sylvian parietal temporal area.^94^ While in stroke patients, the three functions are normally found to be all impaired, in this patient, repetition and spelling from dictation, but not reading, were outside the normal range. This functional dissociation was matched to an anatomical dissociation between a damaged part of the left arcuate fasciculus, originating in the superior temporal gyrus, and a spared part of the left arcuate fasciculus originating in the middle temporal gyrus. This occurs because, compared with vascular lesions of stroke patients, gliomas tend to be smaller and produce cognitive impairments usually less severe and more selective.^97^

### Clinical implications

There is evidence that the neuropsychological status influences patients’ post-operative outcome and their quality of life.^98^ Some authors argue that also pre-operative cognitive can play a role in survival prediction. For instance, Van Kessel *et al*.^99^ tested whether pre-operative cognitive functioning represents an added value in survival prediction obtained by calculating the Cox proportional hazards regression models in high grade glioma (HGG) patients. These authors found that, in addition to the expected predictors of survival, like histomolecular classification, age, the extent of resection, preoperative tumour volume, and Karnofsky performance status (KPS), patients’ memory deficits were of additional prognostic value in HGG. Understanding the channels through which the factors operate that help maintain a functional neuropsychological status and how clinicians can strengthen them is a challenge. Our findings have thus implications for clinical management and can prompt new work to develop personalized approaches to patient care. The effect of CR can influence the pre-surgical planning: patients with high CR are highly preserved at the pre-surgical neuropsychological evaluation: it is likely that they will be candidate to awake surgery (compromised patients, depending on their degree of impairments can be discarded from the awake procedure).^100,101^ Second, the surgeon is more prone to maximize the extent of resection patients resulting preserved at the pre-surgical neuropsychological evaluation. This, in turn, impacts on patients’ survival, as the larger the resection, the longer the patient’s survival.^102–105^

## Conclusion

Brain tumours may inflict devastating effects on patients’ cognitive functions. These effects considerably differ from patient to patient, and this paper shows that patients’ demographic and social background, such as their level of education, the occupational status, and whether they live in a large city or in a rural environment, contributes to account for the extent of the severity of their cognitive deficit. Thus, quantifying the influence of such factors opens up towards developing prevention and personalized rehabilitative interventions.

## Supplementary Material

fcad198_Supplementary_Data

## Data Availability

Both the anonymized data and the command lines to perform the statistical analysis are available from the corresponding author upon request. The results are obtain using Stata. This is a statistical package, available commercially, and routinely purchased by research institutions. It is used as a teaching tool in most postgraduate courses with a statistical analysis component.
