# Supplementary material for: Cognitive reserve and individual differences in brain tumour patients
Source: Brain Commun. 2023 Jul 8;5(4):fcad198. doi: 10.1093/braincomms/fcad198 (PMC10361024; doi:10.1093/braincomms/fcad198)
Supplement: fcad198_Supplementary_Data [file fcad198_supplementary_data.pdf]

Supplementary Material.

In this supplementary material, we report some additional results and description of the data.

In Supplementary Table 1, we report separately the average of the variables for patients included in the regression sample and for those excluded due to missing information for some variables. It is apparent, from the reported t-tests that there are only a small number of statistically significant differences.

Supplementary Table 2 reports the x, y, and z spatial coordinates, according to the Montreal Neurological Institute space system, of the brain areas listed in the first column, with the worst damage, as evidenced by the percentage lesion overlay indicating the percentage of patients having a lesion maximally localized in the listed area. The graphical representation of Table 2 is Figure 1, showing that areas with the worst damage, were the left insula, superior and middle temporal gyrus (and temporal pole), hippocampus and the right insula, rolandic operculum, and the white matter beneath.

Supplementary Tables 3 and 4 present the same regressions reported in Supplementary Tables 4 and 5, respectively but including the coefficients for all the variables which we used to predict the cognitive outcomes, namely the location and type of the tumour, age gender and handedness, the area of Italy, and the date when the MRI and the tests took place. Recall that Supplementary Table 4 shows that the type and location of the tumor does not correlate with the IQ of the patient, as measured by the RCPM test; the only exception to this are a negative correlation for patients affected in the parietal area of the brain, and a positive one for those who have relapsed. We interpret the lack of systematic plausible correlations as an indication that had the test been performed prior to the onset of the tumor similar scores would have obtained.

The final two tables in the appendix, corresponds to Supplementary Table 4

with small changes in the econometric specification: the full tables of coefficients Supplementary Table 6 differs only in the inclusion of a triple interaction term, CR × Volume × Right Hemisphere, and Supplementary Table 5 include separately the three components of CR, education, occupational attainment and status, and the nature of the urban environment.

Supplementary Table 1: Testing for difference in the patients excluded.

Variable	Sample	Non-sample	Diff	p-value
STM	5.24	5.125	.115	.29455
WM	3.918	3.913	.005	.49181
TMT-A	41.568	59.125	-17.557***	.00981
TMT-B	119.311	132.2	-12.889	.24963
TMT-B-A	79.218	79.667	-.449	.48801
Fluency	29.37	23.333	6.037**	.02176
Comprehension	32.335	30.017	2.318**	.0207
Naming	27.063	25.194	1.868**	.02897
Construct	13.014	12.524	.49	.15085
Clock	8.944	9.324	-.379	.23432
Cancel	52.331	48.056	4.276***	.00019
RCPM	28.954	27.286	1.668	.25432
Age	46.051	52.252	-6.201***	.00041
Education	12.429	10.333	2.097***	.00001
Volume	52.488	44.658	7.831	.09439
Observations	673	70		

***Note*:** Stars after the mean indicate a larger value at a statistically significant level, *** $p < 0.01$, ** $p < 0.05$, * $p < 0.1$. Volume is measured in mm^3. STM: short term memory. WM: working memory. TMT: trail making test. Compr: verbal comprehension. Constr: Constructional Apraxia. Naming: noun naming. Cancel: cancellation test.

Supplementary Table 2: Montreal Neurologic Institute spatial coordinates

Area	x	y	z	% lesion overlay
**Patients with LH lesions**				
Insula	-40	0	-9	24,93
Superior Temporal Gyrus	-43	-1	-10	23,67
Temporal Pole	-43	3	-10	23,17
Hippocampus	-37	-7	-18	22,41
Amygdala	-32	-2	-12	21,66
Middle Temporal Gyrus	-46	1	-16	19,64
Fusiform Gyrus	-40	-12	-22	18,38
ParaHippocampal Gyrus	-31	-4	-26	18,38
Inferior Frontal Gyrus	-43	11	5	18,63
Rolandic Operculum	-51	7	-1	18,63
Heschls Gyrus	-43	-18	5	18,13
Inferior Temporal Gyrus	-45	-14	-21	18,13
External capsule	-34	0	-7	24,18
Uncinate fasciculus	-36	-2	-17	24,18
Sagittal stratum (IFOF+ILF)	-37	-9	-16	21,66
Fornix	-34	-11	-16	20,65
Superior longitudinal fasciculus	-31	8	21	16,37
**Patients with RH lesions**				
Insula	38	-7	-1	23,07
Putamen	32	-8	-1	23,07
Heschls Gyrus	38	-17	9	22,26
Rolandic Operculum	37	-22	19	20,24
Superior Temporal Gyrus	40	-15	1	20,64
External capsule	37	-5	-2	23,48
Posterior limb of internal capsule	29	-21	10	21,05
Superior longitudinal fasciculus	35	-18	24	21,05

***Note*:** The Montreal Neurological Institute space system spatial coordinates for the patients in the sample. LH=left hemisphere; RH=right hemisphere; IFOF=inferior fronto occipital fasciculus; ILF=inferior longitudinal fasciculus

Supplementary Table 3: Determinants of RCPM test.

Base Level	-0.745***		
	(0.210)		
Right hemisphere	0.226	Fronto-Parietal	0.349
	(0.267)		(0.217)
Education (years)	0.0614***	Fronto-Temporal	0.119
	(0.0142)		(0.114)
Extra effect for RH	-0.0293	Occipital/P/T	-0.169
	(0.0204)		(0.191)
Professional	0.158	Parietal	-0.224*
	(0.145)		(0.117)
Administrative	-0.0768	Temporal	-0.00763
	(0.107)		(0.109)
Manual unskilled	-0.258**	Temporo-Parietal	-0.162
	(0.116)		(0.167)
Pensioners	0.191	Lefthanded	0.228
	(0.164)		(0.173)
Volume (in log)	-0.0345	Large city	0.00847
	(0.0352)		(0.151)
High-grade Glioma	-0.0310	Smaller city	0.0305
	(0.0939)		(0.0805)
Metastasis	-0.223	Center Italy	0.0901
	(0.246)		(0.127)
Meningioma	-0.0863	South and Islands	-0.0559
	(0.150)		(0.104)
Cavernoma/MAV	-0.117	Female	-0.122
	(0.159)		(0.0755)
Other	-0.0422	Age	0.00572
	(0.253)		(0.0162)
Relapsed	0.202**	Age^2	-0.000199
	(0.0907)		(0.000174)
		Observations	673
		R-squared	0.150

***Note*:** *** $p < 0.01$, ** $p < 0.05$, * $p < 0.1$. Standard errors in parentheses. Result of a OLS regression with the RCPM (normalised to have zero mean and unit standard deviation) as a dependant variable, and the variables used in the main analysis as independent variables. This is intended to detect correlation between the the outcome of the RCPM test and the clinical variables characterising the brain tumor.

Supplementary Table 4: Cognitive reserve and cognitive functionality: the complete regression.

VARIABLES	(1) WM	(2) STM	(3) Fluency	(4) TMT-A	(5) TMT-B	(6) TMT-BA	(7) Compr	(8) Naming	(9) Construct	(10) Clock	(11) Cancel
Base Level	3.860***	5.590***	33.45***	34.38***	102.7***	69.16***	33.91***	28.65***	14.06***	9.488***	53.64***
	(0.134)	(0.144)	(1.776)	(4.393)	(11.35)	(9.552)	(0.757)	(0.737)	(0.379)	(0.422)	(0.929)
Right hemisphere	0.489***	-0.273***	-4.774***	-2.181	-14.59**	-13.77**					
	(0.0913)	(0.0984)	(1.710)	(2.823)	(7.338)	(6.192)					
Cognitive Reserve	0.230***	0.0872*	2.584***	-3.009	-11.67**	-9.177**	0.558**	0.410*	-0.00259	0.331**	-0.254
	(0.0464)	(0.0507)	(0.587)	(1.855)	(4.802)	(4.051)	(0.233)	(0.226)	(0.129)	(0.139)	(0.306)
Extra effect for RH	-0.146*	0.0390	-1.959	2.372	3.976	2.580					
	(0.0781)	(0.0844)	(1.343)	(2.228)	(5.847)	(4.923)					
Volume (in log)	-0.167***	-0.189***	-4.122***	3.955***	12.94***	9.479***	-1.296***	-1.129***	-0.197*	-0.311***	-0.482*
	(0.0411)	(0.0441)	(0.607)	(1.157)	(3.058)	(2.576)	(0.254)	(0.244)	(0.115)	(0.119)	(0.278)
Volume x CR	-0.0110	0.00355	0.868*	-1.854*	-0.878	1.023	0.391*	0.264	0.0577	0.236**	0.0124
	(0.0370)	(0.0400)	(0.495)	(1.041)	(2.707)	(2.279)	(0.204)	(0.198)	(0.107)	(0.112)	(0.265)
Raven score	0.0415***	0.0409***	0.252***	-1.609***	-3.673***	-2.460***	0.165***	0.141***	0.0691***	0.0580**	0.0110
	(0.00851)	(0.00887)	(0.0934)	(0.250)	(0.666)	(0.561)	(0.0446)	(0.0422)	(0.0198)	(0.0233)	(0.0434)
High-grade Glioma	-0.352***	-0.404***	-6.435***	5.276	24.28***	19.64***	-3.017***	-2.420***	-0.553*	-0.108	-0.909
	(0.108)	(0.116)	(1.515)	(3.296)	(8.511)	(7.185)	(0.636)	(0.621)	(0.321)	(0.344)	(0.782)
Metastasis	-0.178	0.0914	4.455	31.93***	23.37	-9.024	-0.748	1.499	-2.172**	-0.809	-2.411
	(0.261)	(0.289)	(4.385)	(8.829)	(24.01)	(20.21)	(1.554)	(1.531)	(0.931)	(0.968)	(2.801)
Meningioma	0.156	0.0277	-1.398	4.618	12.26	7.996	-1.348	-0.180	-0.211	0.398	-0.219
	(0.170)	(0.185)	(2.363)	(4.642)	(12.12)	(10.20)	(1.064)	(0.993)	(0.499)	(0.529)	(1.247)
Cavernoma/MAV	-0.00757	-0.300	-7.120***	18.19***	41.16***	26.45**	-0.607	-0.244	-0.0346	-1.128*	-1.911
	(0.185)	(0.205)	(2.631)	(5.094)	(13.27)	(11.17)	(1.124)	(1.060)	(0.559)	(0.582)	(1.301)
Other	-0.117	-0.146	-7.897**	-2.015	-14.91	-11.88	-1.640	-1.810	-0.216	-2.021*	-0.615
	(0.277)	(0.297)	(3.868)	(10.90)	(27.85)	(23.45)	(1.551)	(1.527)	(1.026)	(1.059)	(3.946)
Relapsed	0.0698	-0.0726	-0.956	1.494	7.115	6.001	0.628	0.644	-0.284	0.0203	-0.164
	(0.104)	(0.112)	(1.453)	(3.281)	(8.456)	(7.123)	(0.626)	(0.608)	(0.307)	(0.334)	(0.750)
Fronto-Parietal	-0.231	-0.351	-0.670	13.02**	1.911	-4.947	-1.625	0.503	-1.440***	-0.576	-2.349*
	(0.271)	(0.291)	(4.147)	(6.433)	(16.98)	(14.30)	(2.266)	(2.047)	(0.546)	(0.603)	(1.312)
Fronto-Temporal	0.214	-0.0200	-0.849	0.677	-4.625	-4.926	-0.248	-1.289*	-0.0645	-0.559	-1.685*
	(0.134)	(0.144)	(1.846)	(4.298)	(11.20)	(9.439)	(0.776)	(0.762)	(0.373)	(0.403)	(0.923)
Occipital/P/T	0.185	-0.0297	0.208	4.721	37.09**	31.30**	-0.435	0.295	-1.340**	-0.857	-1.103
	(0.216)	(0.235)	(3.147)	(5.875)	(15.01)	(12.65)	(1.526)	(1.396)	(0.558)	(0.595)	(1.429)
Parietal	-0.0388	-0.0529	1.320	1.967	14.46	12.28	-0.276	-0.0532	-0.467	-0.865**	-0.670
	(0.136)	(0.146)	(1.896)	(3.542)	(9.205)	(7.755)	(0.850)	(0.812)	(0.373)	(0.399)	(0.912)
Temporal	0.0219	0.0715	0.214	4.115	21.64	18.09	-1.381**	-1.994***	-0.323	-0.737	-0.649
	(0.121)	(0.131)	(1.690)	(5.574)	(14.24)	(12.03)	(0.677)	(0.661)	(0.478)	(0.489)	(1.129)
Temporo-Parietal	0.334*	0.0450	-0.448	-3.063	1.736	4.478	-2.275**	-2.332**	-1.289**	0.347	-1.907
	(0.196)	(0.204)	(2.681)	(6.286)	(16.08)	(13.54)	(1.157)	(1.114)	(0.565)	(0.609)	(1.182)
Lefthanded	-0.479**	-0.239	1.822	-7.010	-11.36	-7.070	-0.0276	0.548	-0.514	0.882	1.857
	(0.191)	(0.212)	(2.901)	(5.461)	(13.95)	(12.08)	(1.250)	(1.200)	(0.562)	(0.597)	(1.664)
Center Italy	-0.101	-0.121	2.825	0.0181	-1.502	-2.187	0.169	0.763	0.201	-0.468	0.791
	(0.146)	(0.158)	(1.917)	(5.017)	(12.81)	(10.79)	(0.788)	(0.775)	(0.486)	(0.552)	(1.223)
South and Islands	-0.133	0.0283	-1.642	7.391**	13.26	6.812	-0.280	-0.762	-0.402	-0.101	-0.123
	(0.112)	(0.121)	(1.618)	(3.311)	(8.520)	(7.184)	(0.716)	(0.693)	(0.326)	(0.344)	(0.763)
Female	-0.195**	-0.0843	1.512	3.963	11.45*	7.156	0.205	-0.389	-0.250	-0.493*	-0.294
	(0.0868)	(0.0937)	(1.193)	(2.650)	(6.918)	(5.846)	(0.508)	(0.492)	(0.265)	(0.285)	(0.630)
Age	-0.0421**	-0.0318	-0.00815	-0.424	-0.178	0.244	0.0566	0.243**	0.112**	0.0425	0.0689
	(0.0180)	(0.0194)	(0.255)	(0.584)	(1.518)	(1.278)	(0.107)	(0.104)	(0.0568)	(0.0625)	(0.155)
Age^2	0.000242	0.000221	-0.00117	0.00945	0.0139	0.00407	-0.00119	-0.00269**	-0.00142**	-0.000645	-0.00142
	(0.000189)	(0.000204)	(0.00272)	(0.00602)	(0.0157)	(0.0132)	(0.00113)	(0.00110)	(0.000595)	(0.000658)	(0.00162)
Date of test	0.0627***	0.0357*	-0.348	-0.369	-1.983	-1.537	-0.0708	-0.106	-0.0818	0.0469	-0.107
	(0.0190)	(0.0198)	(0.240)	(0.552)	(1.427)	(1.202)	(0.0985)	(0.0957)	(0.0507)	(0.0557)	(0.183)
Observations	538	571	459	354	341	340	400	416	250	233	169
R-squared	0.355	0.206	0.295	0.366	0.376	0.300	0.306	0.236	0.249	0.233	0.166

***Note*:** Standard errors in parentheses. *** $p < 0.01$, ** $p < 0.05$, * $p < 0.1$. This Supplementary Table reports in full the regression from which Table 5 in the main text is derived. The base level (top row) is the predicted value of a right-handed male patient of average age and education, with the average RCPM score, at the first surgery for a frontal low grade glioma tumor in the left hemisphere, with the average log-volume, who is employed as a skilled manual worker or equivalent occupation and lives in a town in the north of Italy with fewer than 55,000 inhabitants, and whose MRI and tests took place on 31 January 2015.

Supplementary Table 5: Separate components of cognitive reserve and cognitive functionality. Part I

VARIABLES	(1) WM	(2) MBT	(3) fluency	(4) TMTA	(5) TMTB	(6) TM-B-A	(7) compr	(8) namingN	(9) construct	(10) orologio	(11) neglect
Base Value	3.769***	5.378***	31.45***	40.68***	110.5***	66.52***	33.52***	28.48***	13.84***	9.231***	52.80***
	(0.172)	(0.184)	(2.267)	(6.275)	(17.13)	(14.47)	(0.926)	(0.896)	(0.477)	(0.527)	(1.211)
Right hemisphere	0.404*	0.0288	-7.374*	4.705	-4.977	-4.484					
	(0.213)	(0.224)	(3.932)	(5.922)	(16.36)	(13.82)					
Education (in years)	0.0476***	0.0329*	0.540**	-0.751	-1.948	-1.517	0.146*	0.0473	0.0908**	0.125***	-0.0804
	(0.0171)	(0.0184)	(0.217)	(0.636)	(1.713)	(1.454)	(0.0847)	(0.0816)	(0.0442)	(0.0477)	(0.106)
Extra effect for RH	-0.0470	0.00497	-0.567	1.366*	2.681	2.195					
	(0.0303)	(0.0324)	(0.527)	(0.828)	(2.277)	(1.926)					
Professional	0.152	0.0838	5.656**	-6.488	0.269	6.866	1.807*	1.454	-0.508	-0.414	0.184
	(0.201)	(0.219)	(2.602)	(8.454)	(22.47)	(18.98)	(1.018)	(0.984)	(0.494)	(0.527)	(1.244)
Extra effect for RH	-0.135	-0.175	2.205	0.653	-17.04	-23.84					
	(0.345)	(0.367)	(6.240)	(10.37)	(28.07)	(23.70)					
Administrative	0.117	0.0322	2.061	-9.566	-4.434	6.150	0.191	0.798	0.0632	0.274	0.635
	(0.151)	(0.161)	(1.888)	(6.034)	(16.25)	(13.78)	(0.729)	(0.705)	(0.362)	(0.389)	(0.912)
Extra effect for RH	0.220	0.0650	3.194	-3.938	-17.13	-18.38					
	(0.256)	(0.273)	(4.764)	(7.474)	(20.37)	(17.26)					
Manual unskilled	-0.197	0.156	0.980	0.504	2.140	6.666	0.0735	-0.465	0.415	0.612	1.159
	(0.159)	(0.171)	(1.984)	(6.357)	(17.44)	(14.73)	(0.763)	(0.729)	(0.446)	(0.500)	(1.111)
Extra effect for RH	0.135	-0.208	5.468	-3.289	6.626	2.308					
	(0.323)	(0.347)	(4.881)	(8.859)	(23.96)	(20.24)					
Pensioners	-0.156	0.357	-0.150	-17.32**	11.72	29.67*	0.458	-0.0983	1.043*	-0.416	0.608
	(0.206)	(0.222)	(2.789)	(7.173)	(19.44)	(16.42)	(1.090)	(1.042)	(0.588)	(0.624)	(1.499)
Extra effect for RH	0.120	-0.746**	5.207	-1.286	-18.75	-20.62					
	(0.338)	(0.366)	(9.730)	(9.078)	(24.78)	(20.93)					
Large city	0.444**	0.694***	4.135	3.644	-8.524	-10.14	0.707	0.239	0.232	0.358	1.644
	(0.206)	(0.221)	(2.514)	(9.377)	(24.67)	(20.87)	(0.998)	(0.972)	(0.567)	(0.644)	(1.664)
Extra effect for RH	-0.300	-0.719*	-14.34*	-11.08	-29.42	-15.12					
	(0.405)	(0.428)	(8.436)	(12.03)	(31.88)	(26.95)					
Smaller city	0.128	0.252**	0.133	4.538	-10.25	-8.353	-0.409	-0.207	-0.213	0.148	0.112
	(0.115)	(0.124)	(1.470)	(4.785)	(13.09)	(11.10)	(0.569)	(0.544)	(0.265)	(0.282)	(0.658)
Extra effect for RH	0.0120	-0.307	1.569	-10.60*	2.451	6.158					
	(0.189)	(0.202)	(3.428)	(5.693)	(15.42)	(13.05)					
Volume (in log)	-0.226***	-0.253***	-5.188***	4.662**	12.80**	8.732	-0.240	-0.150	-0.298	-0.453*	-0.765
	(0.0874)	(0.0941)	(1.263)	(2.321)	(6.310)	(5.329)	(0.549)	(0.528)	(0.244)	(0.256)	(0.601)
Volume x Education	-0.0240*	-0.00558	-0.372*	-0.0333	0.885	0.955	-0.00784	-0.169**	0.0634*	0.0287	0.0148
	(0.0134)	(0.0146)	(0.190)	(0.378)	(1.030)	(0.869)	(0.0834)	(0.0798)	(0.0368)	(0.0393)	(0.0928)
Extra effect for Prof.	-0.0671	-0.108	0.450	-4.324	5.733	8.431	0.0384	0.198	-0.557	-0.160	-1.672
	(0.145)	(0.157)	(2.157)	(4.274)	(11.78)	(9.950)	(0.905)	(0.871)	(0.429)	(0.452)	(1.090)
Extra effect for Admin.	0.0970	0.200*	2.912*	-2.297	1.432	2.233	-0.575	-0.0528	-0.0367	-0.0477	0.684
	(0.104)	(0.111)	(1.557)	(2.880)	(8.027)	(6.779)	(0.654)	(0.633)	(0.279)	(0.296)	(0.714)
Extra effect for Manual unsk.	0.0752	0.104	-0.775	5.586	15.77*	9.955	-1.113	-1.310*	0.320	-0.349	0.0697
	(0.125)	(0.136)	(1.709)	(3.405)	(9.110)	(7.694)	(0.779)	(0.740)	(0.343)	(0.371)	(0.833)
Extra effect for Pens.	-0.202	-0.126	-4.447*	-2.216	-0.732	2.535	-3.394***	-4.424***	0.782	0.274	-0.590
	(0.174)	(0.187)	(2.584)	(5.577)	(15.62)	(13.20)	(1.036)	(0.998)	(0.675)	(0.700)	(1.652)
Extra effect in large city	0.124	-0.0995	3.503*	5.316	-13.58	-14.98	0.664	0.804	0.347	0.689	-1.009
	(0.160)	(0.171)	(2.065)	(4.899)	(13.45)	(11.36)	(0.832)	(0.807)	(0.437)	(0.480)	(1.328)
Extra effect in small city	0.0931	-0.0243	0.872	-1.639	-8.253	-5.589	-0.848*	-0.825*	0.146	0.514**	0.361
	(0.0799)	(0.0864)	(1.239)	(2.237)	(6.006)	(5.073)	(0.500)	(0.476)	(0.218)	(0.230)	(0.555)

***Note*:** Supplementary Table continues in the next page.

Table 5 (ctd.): Separate components of cognitive reserve and cognitive functionality. Part II

VARIABLES	(1)<br>WM	(2)<br>MBT	(3)<br>fluency	(4)<br>TMTA	(5)<br>TMTB	(6)<br>TM-B-A	(7)<br>compr	(8)<br>namingN	(9)<br>construct	(10)<br>orologio	(11)<br>neglect
Raven score	0.0419***	0.0343***	0.265***	-1.625***	-3.966***	-2.668***	0.146***	0.138***	0.0671***	0.0643***	0.0357
	(0.00886)	(0.00914)	(0.0993)	(0.257)	(0.703)	(0.594)	(0.0464)	(0.0434)	(0.0201)	(0.0239)	(0.0461)
High-grade Glioma	-0.364***	-0.390***	-6.320***	4.158	22.78***	19.30***	-3.104***	-2.617***	-0.434	-0.0710	-0.588
	(0.110)	(0.117)	(1.540)	(3.269)	(8.681)	(7.348)	(0.648)	(0.625)	(0.322)	(0.347)	(0.830)
Metastasis	-0.178	0.174	4.617	28.99***	20.29	-8.260	-0.686	1.699	-1.997**	-0.823	-2.463
	(0.265)	(0.291)	(4.418)	(8.879)	(24.49)	(20.68)	(1.568)	(1.529)	(0.934)	(0.975)	(2.957)
Meningioma	0.197	0.0451	0.126	3.460	10.35	6.913	-1.026	0.286	-0.296	0.501	0.425
	(0.175)	(0.189)	(2.431)	(4.769)	(12.79)	(10.80)	(1.071)	(0.994)	(0.518)	(0.551)	(1.330)
Cavernoma/MAV	0.0331	-0.225	-6.306**	16.05***	41.33***	27.88**	-0.492	-0.139	0.125	-1.382**	-1.577
	(0.189)	(0.208)	(2.662)	(5.168)	(13.75)	(11.61)	(1.119)	(1.046)	(0.570)	(0.601)	(1.378)
Other	-0.109	-0.115	-6.799*	-6.175	-19.46	-11.79	-1.969	-2.114	-0.470	-1.803*	0.396
	(0.280)	(0.297)	(3.919)	(10.95)	(28.89)	(24.40)	(1.572)	(1.530)	(1.047)	(1.083)	(4.596)
Relapsed	0.0665	-0.0803	-1.071	0.606	5.049	5.466	0.850	0.730	-0.102	0.167	-0.270
	(0.105)	(0.112)	(1.466)	(3.279)	(8.691)	(7.345)	(0.627)	(0.603)	(0.306)	(0.336)	(0.778)
Fronto-Parietal	-0.120	-0.234	-1.793	14.15**	-1.276	-8.779	-1.677	0.0621	-1.632***	-0.412	-2.318*
	(0.276)	(0.292)	(4.205)	(6.373)	(17.37)	(14.67)	(2.260)	(2.028)	(0.550)	(0.609)	(1.356)
Fronto-Temporal	0.214	-0.0178	-0.494	0.510	-2.787	-2.279	-0.176	-1.270*	0.0364	-0.559	-1.435
	(0.135)	(0.145)	(1.868)	(4.266)	(11.47)	(9.704)	(0.774)	(0.752)	(0.372)	(0.404)	(0.938)
Occipital/P/T	0.196	-0.0267	0.0911	3.566	33.41**	28.87**	-0.424	0.294	-1.356**	-0.903	-0.639
	(0.219)	(0.237)	(3.182)	(5.919)	(15.59)	(13.17)	(1.524)	(1.390)	(0.559)	(0.599)	(1.497)
Parietal	-0.0236	-0.0754	0.595	2.785	12.92	10.67	-0.624	-0.209	-0.505	-0.809**	-0.872
	(0.138)	(0.147)	(1.933)	(3.516)	(9.440)	(7.978)	(0.857)	(0.810)	(0.370)	(0.401)	(0.934)
Temporal	0.0332	0.0416	-0.531	0.663	20.68	18.96	-1.642**	-2.230***	-0.445	-0.829	0.229
	(0.123)	(0.133)	(1.718)	(5.668)	(14.94)	(12.67)	(0.688)	(0.663)	(0.493)	(0.507)	(1.217)
Temporo-Parietal	0.338*	0.0581	-0.0608	-4.352	9.006	11.98	-1.893	-1.910*	-1.209**	0.148	-1.813
	(0.199)	(0.204)	(2.740)	(6.369)	(16.84)	(14.22)	(1.160)	(1.102)	(0.579)	(0.619)	(1.257)
Lefthanded	-0.468**	-0.193	2.277	-3.944	-12.07	-9.736	0.377	0.876	-0.615	0.898	1.404
	(0.194)	(0.213)	(2.926)	(5.496)	(14.44)	(12.55)	(1.254)	(1.191)	(0.576)	(0.610)	(1.741)
Center Italy	-0.111	-0.166	2.464	-2.171	-9.839	-7.231	0.0504	0.500	0.388	-0.157	1.190
	(0.151)	(0.161)	(1.951)	(5.175)	(13.62)	(11.50)	(0.796)	(0.775)	(0.497)	(0.561)	(1.275)
South and Islands	-0.147	-0.0310	-1.869	7.060**	12.33	5.846	-0.492	-1.083	-0.321	0.0984	-0.118
	(0.120)	(0.128)	(1.721)	(3.511)	(9.326)	(7.877)	(0.759)	(0.728)	(0.336)	(0.356)	(0.797)
Female	-0.240***	-0.110	1.320	4.939*	15.49**	9.827	0.233	-0.508	-0.265	-0.676**	-0.432
	(0.0888)	(0.0949)	(1.219)	(2.654)	(7.145)	(6.051)	(0.514)	(0.494)	(0.267)	(0.287)	(0.670)
Age	-0.0417**	-0.0306	-0.0131	-0.984*	0.0791	1.003	0.0354	0.208**	0.129**	0.0489	0.0469
	(0.0186)	(0.0198)	(0.261)	(0.596)	(1.597)	(1.349)	(0.108)	(0.105)	(0.0585)	(0.0647)	(0.167)
Age^2	0.000239	0.000200	-0.00133	0.0171***	0.0118	-0.00478	-0.00111	-0.00243**	-0.00161**	-0.000654	-0.00120
	(0.000199)	(0.000212)	(0.00284)	(0.00628)	(0.0168)	(0.0142)	(0.00116)	(0.00112)	(0.000624)	(0.000693)	(0.00176)
Date of test	0.0600***	0.0282	-0.418*	-0.326	-2.009	-1.461	-0.0790	-0.129	-0.0581	0.0705	-0.178
	(0.0193)	(0.0200)	(0.244)	(0.553)	(1.477)	(1.248)	(0.0992)	(0.0953)	(0.0520)	(0.0569)	(0.198)
Observations	538	571	459	354	341	340	400	416	250	233	169
R-squared	0.380	0.247	0.331	0.430	0.409	0.332	0.341	0.287	0.315	0.300	0.231

Note: Standard errors in parentheses. *** $p < 0.01$, ** $p < 0.05$, * $p < 0.1$. In this table the three components of cognitive reserve, and the respective interaction terms with the hemisphere and the volume of the lesion are entered separately as explanatory variables. The base level (top row) and all the other variables are as in Supplementary Table 4.

Supplementary Table 6: Triple interaction: add CR × Volume × Right Hemisphere.

VARIABLES	(1)<br>WM	(2)<br>STM	(3)<br>Fluency	(4)<br>TMT-A	(5)<br>TMT-B	(6)<br>TMT-B-A	(7)<br>Compr	(8)<br>Naming	(9)<br>Construct	(10)<br>Clock	(11)<br>Cancel
Base Level	3.854***	5.588***	33.19***	34.56***	101.9***	68.37***	33.91***	28.65***	14.06***	9.488***	53.64***
	(0.134)	(0.144)	(1.773)	(4.400)	(11.34)	(9.555)	(0.757)	(0.737)	(0.379)	(0.422)	(0.929)
Right hemisphere	0.496***	-0.265***	-4.786***	-2.641	-14.67**	-13.45**					
	(0.0913)	(0.0982)	(1.716)	(2.844)	(7.378)	(6.232)					
Cognitive Reserve	0.231***	0.0867*	2.586***	-2.832	-10.95**	-8.625**	0.558**	0.410*	-0.00259	0.331**	-0.254
	(0.0464)	(0.0507)	(0.583)	(1.857)	(4.808)	(4.058)	(0.233)	(0.226)	(0.129)	(0.139)	(0.306)
Extra effect for RH	-0.153*	0.0377	-2.223	2.270	3.404	2.123					
	(0.0783)	(0.0849)	(1.407)	(2.227)	(5.843)	(4.923)					
Volume (in log)	-0.226***	-0.263***	-4.870***	5.993***	14.83***	9.213**	-1.296***	-1.129***	-0.197*	-0.311***	-0.482*
	(0.0518)	(0.0561)	(0.662)	(1.920)	(5.237)	(4.414)	(0.254)	(0.244)	(0.115)	(0.119)	(0.278)
Extra effect for RH	0.143*	0.179**	3.819***	-2.857	-1.814	1.105					
	(0.0759)	(0.0822)	(1.447)	(2.300)	(6.146)	(5.180)					
Volume x CR	-0.0131	0.0205	1.054**	-0.366	5.591	6.064	0.391*	0.264	0.0577	0.236**	0.0124
	(0.0461)	(0.0500)	(0.523)	(1.700)	(4.461)	(3.759)	(0.204)	(0.198)	(0.107)	(0.112)	(0.265)
Extra effect for RH	-0.00368	-0.0614	-0.570	-2.080	-10.05*	-8.055*					
	(0.0782)	(0.0836)	(1.549)	(2.145)	(5.621)	(4.737)					
Raven score	0.0418***	0.0408***	0.262***	-1.628***	-3.718***	-2.489***	0.165***	0.141***	0.0691***	0.0580**	0.0110
	(0.00852)	(0.00888)	(0.0929)	(0.250)	(0.665)	(0.560)	(0.0446)	(0.0422)	(0.0198)	(0.0233)	(0.0434)
High-grade Glioma	-0.365***	-0.420***	-6.308***	5.444*	24.17***	19.31***	-3.017***	-2.420***	-0.553*	-0.108	-0.909
	(0.108)	(0.116)	(1.508)	(3.299)	(8.509)	(7.190)	(0.636)	(0.621)	(0.321)	(0.344)	(0.782)
Metastasis	-0.210	0.0474	4.504	32.78***	21.97	-10.03	-0.748	1.499	-2.172**	-0.809	-2.411
	(0.261)	(0.289)	(4.358)	(8.855)	(23.97)	(20.19)	(1.554)	(1.531)	(0.931)	(0.968)	(2.801)
Meningioma	0.160	0.0333	-0.908	4.336	11.67	7.794	-1.348	-0.180	-0.211	0.398	-0.219
	(0.170)	(0.184)	(2.355)	(4.642)	(12.11)	(10.20)	(1.064)	(0.993)	(0.499)	(0.529)	(1.247)
Cavernoma/MAV	-0.0299	-0.321	-6.831***	18.25***	41.22***	26.45**	-0.607	-0.244	-0.0346	-1.128*	-1.911
	(0.185)	(0.205)	(2.623)	(5.089)	(13.24)	(11.16)	(1.124)	(1.060)	(0.559)	(0.582)	(1.301)
Other	-0.124	-0.152	-7.410*	-2.358	-14.07	-10.69	-1.640	-1.810	-0.216	-2.021*	-0.615
	(0.277)	(0.297)	(3.852)	(10.91)	(27.82)	(23.44)	(1.551)	(1.527)	(1.026)	(1.059)	(3.946)
Relapsed	0.0844	-0.0592	-0.844	1.425	7.649	6.546	0.628	0.644	-0.284	0.0203	-0.164
	(0.104)	(0.112)	(1.445)	(3.284)	(8.451)	(7.124)	(0.626)	(0.608)	(0.307)	(0.334)	(0.750)
Fronto-Parietal	-0.251	-0.386	-2.233	13.57**	1.901	-5.416	-1.625	0.503	-1.440***	-0.576	-2.349*
	(0.271)	(0.291)	(4.160)	(6.444)	(16.98)	(14.31)	(2.266)	(2.047)	(0.546)	(0.603)	(1.312)
Fronto-Temporal	0.215	-0.0251	-0.496	1.404	-3.213	-4.225	-0.248	-1.289*	-0.0645	-0.559	-1.685*
	(0.134)	(0.144)	(1.843)	(4.319)	(11.24)	(9.479)	(0.776)	(0.762)	(0.373)	(0.403)	(0.923)
Occipital/P/T	0.171	-0.0521	-0.834	4.906	37.27**	31.28**	-0.435	0.295	-1.340**	-0.857	-1.103
	(0.215)	(0.235)	(3.159)	(5.871)	(14.99)	(12.63)	(1.526)	(1.396)	(0.558)	(0.595)	(1.429)
Parietal	-0.0397	-0.0577	1.283	2.372	15.94*	13.26*	-0.276	-0.0532	-0.467	-0.865**	-0.670
	(0.136)	(0.146)	(1.884)	(3.548)	(9.226)	(7.778)	(0.850)	(0.812)	(0.373)	(0.399)	(0.912)
Temporal	0.0252	0.0687	0.175	4.033	21.90	18.50	-1.381**	-1.994***	-0.323	-0.737	-0.649
	(0.121)	(0.131)	(1.680)	(5.570)	(14.22)	(12.02)	(0.677)	(0.661)	(0.478)	(0.489)	(1.129)
Temporo-Parietal	0.332*	0.0343	-0.140	-3.146	0.640	3.389	-2.275**	-2.332**	-1.289**	0.347	-1.907
	(0.196)	(0.203)	(2.675)	(6.287)	(16.07)	(13.54)	(1.157)	(1.114)	(0.565)	(0.609)	(1.182)
Lefthanded	-0.471**	-0.234	1.766	-7.289	-13.61	-9.196	-0.0276	0.548	-0.514	0.882	1.857
	(0.190)	(0.211)	(2.884)	(5.480)	(14.00)	(12.13)	(1.250)	(1.200)	(0.562)	(0.597)	(1.664)
Center Italy	-0.0939	-0.105	2.690	0.208	-0.110	-0.947	0.169	0.763	0.201	-0.468	0.791
	(0.146)	(0.158)	(1.916)	(5.024)	(12.81)	(10.80)	(0.788)	(0.775)	(0.486)	(0.552)	(1.223)
South and Islands	-0.139	0.0344	-1.633	7.856**	15.30*	8.462	-0.280	-0.762	-0.402	-0.101	-0.123
	(0.113)	(0.122)	(1.609)	(3.341)	(8.577)	(7.239)	(0.716)	(0.693)	(0.326)	(0.344)	(0.763)
Female	-0.185**	-0.0756	1.602	3.993	11.56*	7.064	0.205	-0.389	-0.250	-0.493*	-0.294
	(0.0869)	(0.0936)	(1.188)	(2.648)	(6.913)	(5.847)	(0.508)	(0.492)	(0.265)	(0.285)	(0.630)
Age	-0.0392**	-0.0269	0.0639	-0.431	0.0959	0.532	0.0566	0.243**	0.112**	0.0425	0.0689
	(0.0182)	(0.0195)	(0.254)	(0.589)	(1.535)	(1.293)	(0.107)	(0.104)	(0.0568)	(0.0625)	(0.155)
Age^2	0.000213	0.000174	-0.00191	0.00948	0.0110	0.00113	-0.00119	-0.00269**	-0.00142**	-0.000645	-0.00142
	(0.000191)	(0.000205)	(0.00272)	(0.00606)	(0.0158)	(0.0134)	(0.00113)	(0.00110)	(0.000595)	(0.000658)	(0.00162)
Date of test	0.0606***	0.0333*	-0.354	-0.375	-2.017	-1.576	-0.0708	-0.106	-0.0818	0.0469	-0.107
	(0.0191)	(0.0198)	(0.238)	(0.552)	(1.425)	(1.201)	(0.0985)	(0.0957)	(0.0507)	(0.0557)	(0.183)
Observations	538	571	459	354	341	340	400	416	250	233	169
R-squared	0.355	0.206	0.295	0.366	0.376	0.300	0.306	0.236	0.249	0.233	0.166

Note: Standard errors in parentheses. *** $p < 0.01$, ** $p < 0.05$, * $p < 0.1$. This Supplementary Table is identical to Supplementary Table 4, except for the addition of a triple interaction term, given by the product of CR, the log of the volume of the lesion, and the indicator variable for the right hemisphere.
